# Prospective Evaluation of Specific IgE Profile and Quality-of-Life During Allergen-Specific Immunotherapy with House Dust Mite: A Pilot Study

**DOI:** 10.3390/medicina62010009

**Published:** 2025-12-19

**Authors:** Sandra Sakalauskaite, Ligita Pilkyte, Edita Gasiuniene, Brigita Gradauskiene

**Affiliations:** 1 Department of Immunology and Allergology, Lithuanian University of Health Sciences, 50161 Kaunas, Lithuania; 2 Laboratory of Immunology, Department of Immunology and Allergology, Lithuanian University of Health Sciences, 50161 Kaunas, Lithuania

**Keywords:** allergic airway diseases, ASIT, asthma, BMI, IgE, rhinitis, sensitization, molecular allergology, vitamin D

## Abstract

*Background and Objectives*: The average prevalence of sensitization to house dust mite in developed countries is more than 20%. The three major allergens of *D. pteronyssinus*—Der p 1, Der p 2, and Der p 23—have been associated with asthma severity. Allergen-specific immunotherapy (ASIT) is the only personalized and effective treatment that can change the natural course of allergic diseases such as allergic rhinitis or allergic asthma. Despite ASIT being an established treatment method, its effectiveness is still assessed using patient-reported outcome measures that determine quality of life, and there are no objective biomarkers that can accurately and reliably indicate the therapeutic efficacy of ASIT. This study aimed to monitor sensitization profiles to allergens, assess the effectiveness of ASIT, and evaluate total nasal symptom score (TNSS) and quality of life after six months of ASIT treatment. *Materials and Methods*: The molecular allergy diagnostic system was used to assess changes in patients’ sensitization profiles to allergens, and the validated questionnaires RQLQ and TNSS were used for quality-of-life assessment. *Results*: After 6 months of ASIT treatment against house dust mite allergens, a statistically significant increase in sIgE against the Der p 23 component was noted. In addition, a significant decrease in practical problems and an improvement in patients‘ emotional state were observed, while the TNSS score remained unchanged. *Conclusions*: Continuous monitoring of the Der p 23 component during further stages of ASIT is, therefore, essential to determine whether the observed changes reflect de novo sensitization or represent an immunological response to therapy.

## 1. Introduction

Allergic diseases, such as allergic rhinitis (AR) and allergic asthma (AA), are among the most common chronic conditions related to allergic sensitization. It is estimated that in Europe, one in three individuals suffers from some form of allergic disorder [1]. AR and AA significantly affect patients‘ quality of life, including work performance, sleep quality, and emotional well-being [2,3].

One of the main perennial allergens is house dust mite (HDM). According to the European Community Respiratory Health Survey I, the average prevalence of sensitization to house dust mite in developed countries is 21.7% [4]. The two dominant clinically relevant species of HDM in Europe are *Dermatophagoides pteronyssinus* (*D. pteronyssinus*) and *Dermatophagoides farinae* (*D. farinae*) [5]. The Allergen Nomenclature Subcommittee of the World Health Organization and the International Union of Immune Societies has identified 83 allergenic proteins from HDM, of which the most common are Der p 1, Der p 2, Der p 23, Der f 1, and Der f 2 [6,7]. Major house dust mite allergens Der p 1 and Der p 2 have long been associated with asthma risk and disease severity. Recent component-resolved diagnostics studies indicate that Der p 23 may also be linked to increased asthma severity and possibly to the development of asthma in sensitized individuals, as reported in a Ukrainian study [8]. However, the evidence for Der p 23 is still emerging, and further longitudinal data are needed [9,10].

Treatment of AR consists of allergen avoidance and symptom-relieving medications such as antihistamines and intranasal corticosteroids. Although management appears effective, it does not address the underlying cause of the disease [11]. Allergen-specific immunotherapy (ASIT) is the only personalized and effective treatment that can change the natural course of allergic airway diseases such as allergic rhinitis and allergic asthma. It is based on repeated and controlled administration of allergens and targets the underlying cause of the disease, promotes immune tolerance to specific allergens, and ensures long-term effects [12,13].

The evaluation of the effectiveness of allergen-specific immunotherapy is based on the induction of a tolerogenic state, i.e., suppression of the type 2 immunity response, through the action of cytokines IL-10 and TGF-β secreted by Tregs. Allergen-specific IgG4 antibodies act as “blocking antibodies”, competing with IgE for allergen binding and thereby reducing IgE–allergen complex formation, mast cell and basophil activation, antigen presentation, and the release of type 2 pro-inflammatory cytokines [14,15,16]. Recent studies have shown that vitamin D stimulates the production of Treg cells that secrete IL-10 and modulate B cell differentiation, contributing to suppression of the Th2 inflammatory pathway and reduction of IgE secretion [17]. These findings suggest that adequate vitamin D levels may play a role in remodeling the immune response during ASIT. Furthermore, low vitamin D levels are commonly observed in individuals with higher body mass index (BMI). At the same time, obesity is also associated with chronic low-grade inflammation, which can further influence immune regulation and vitamin D metabolism [18]. Obesity itself can also affect adaptive immune responses and alter T-cell balance. Evidence shows that obese individuals have a higher risk of asthma, and obesity-associated asthma is often more severe and complex to treat. Although obesity generally shifts the Th1/Th2 equilibrium toward a Th1 profile, it may also coexist with type-2-driven asthma phenotypes [19]

Longitudinal analyses have shown that immunoglobulin responses during ASIT follow characteristic patterns. For example, Yang et al. demonstrated that sIgE levels typically rise during the initial up-dosing phase and subsequently decline during maintenance, whereas sIgG4 levels increase steadily throughout treatment. However, these immunoglobulin changes did not correlate consistently with clinical improvement, suggesting that sIgG4 alone is not a reliable biomarker for monitoring therapeutic response to ASIT [20].

Despite ASIT being an established treatment method, its effectiveness is still assessed using patient-reported outcome measures that determine quality of life. Such assessment is not objective and, with the move toward personalized medicine, does not meet the requirements of either clinicians or patients. There are currentlyno objective biomarkers that can accurately and reliably indicate the therapeutic efficacy of ASIT [21].

This study aimed to monitor sensitization profiles to allergens in order to assess the effectiveness of ASIT and evaluate total nasal symptom scores and quality of life after six months of ASIT treatment. By conducting molecular studies, we aim to contribute to the exploration of ASIT mechanisms and to the development of future prognostic models for treatment. It is generally claimed that ASIT reduces the risk of new allergen sensitizations; however, molecular studies have also observed the possible emergence of new sensitizations induced by ASIT [22]. To confirm this, it is necessary to evaluate a wide range of molecular allergen components rather than selecting only a few. Therefore, in our study, we used molecular component analysis to evaluate changes in patients’ allergen sensitization profiles during allergen-specific immunotherapy.

## 2. Materials and Methods

### 2.1. Study Design

A pilot study was performed in the Department of Immunology and Allergology at the Hospital of the Lithuanian University of Health Sciences Kauno Klinikos. The study was approved by the Kaunas Regional Biomedical Research Ethics Committee on 3 February 2025 (No. BE-2-6) and written informed consent was obtained from each participant prior to the study.

Patients with persistent allergic rhinitis, diagnosed according to the Allergic Rhinitis and its Impact on Asthma (ARIA) guidelines [23], and allergic asthma, diagnosed following the Global Initiative for Asthma (GINA) guidelines [24], with symptoms lasting at least two years, were enrolled in the study. All participants were between 18 and 60 years old, and sensitization to HDM was diagnosed by skin prick testing. The main exclusion criteria were clinically significant sensitization to other inhaled aeroallergens, malignant diseases, systemic autoimmune diseases, prior treatment with allergen immunotherapy, pregnancy, and use of vitamin D supplements during the past three months.

### 2.2. Assessment of Symptoms Severity and Quality of Life

All patients were asked to complete the Total Nasal Symptom Score (TNSS) [25] and the Rhinoconjunctivitis Quality of Life Questionnaire (RQLQ), which is the most frequently used, specific, and validated instrument involved in ASIT trials [26,27,28]. Responses were rated on a 7-point Likert scale, and domains and total scores were assessed on a scale from 0 to 6 (0 = not problematic; 6 = very problematic), with lower scores indicating better quality of life. Mean RQLQ scores obtained from each domain at the baseline visit, after the introduction of ASIT, and after the third maintenance dose visits were compared.

### 2.3. Evaluation of Allergic Sensitization

Allergic sensitization was confirmed by a skin prick test before enrollment in the study, according to the standard protocol using standard inhalant allergens (Inmunotec, Madrid, Spain). Blood samples for specific immunoglobulin E (sIgE), total IgE, and vitamin D assays were collected into serum tubes. The concentration of sIgE in serum was determined in vitro using an ELISA-based multiplex allergy test—Allergy Explorer, ALEX2 (Macro Array Diagnostics GmbH, Vienna, Austria). According to the manufacturer‘s instructions, sensitization was defined by detecting an sIgE level greater than 0.3 kUA/L. The dynamics of sIgE concentration was assesed at three points: before treatment (V0); after the introduction of ASIT (V2), and after the third maintenance dose (V5). Vitamin D, measured as serum 25-hydroxyvitamin D (25(OH)D), was evaluated by ELISA using a commercial kit (BioVendor, Brno, Czech Republic). The limit of detection was 2.81 ng/mL.

### 2.4. Treatment by Allergen-Specific Immunotherapy Plan

**Figure 1:**
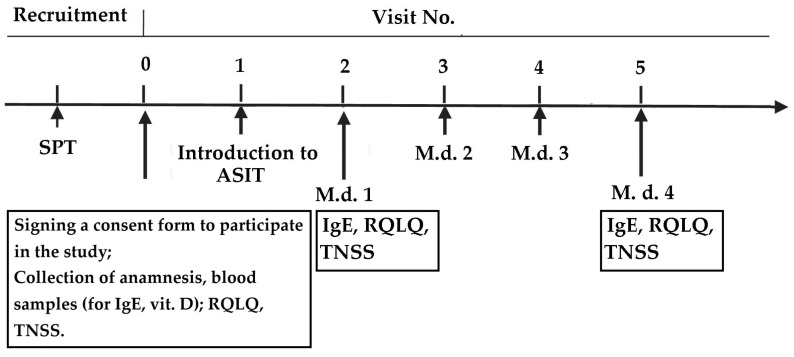
Plan of study visits and procedures performed. Blood samples were collected and questionnaires were completed before drug administration. Abbreviations: IgE—immunoglobulin E; m.d.—maintenance dose; RQLQ—Rhinoconjunctivitis Quality of Life Questionnaire; TNSS—Total Nasal Symptom Score; vit. D—vitamin D.

Allergen-specific immunotherapy was administered using a mixture of *Dermatophagoides pteronyssinus* and *Dermatophagoides farinae* according to the manufacturer’s recommendations (ALXOID, Inmunotec, Spain), using the Rush method. The Rush method uses an allergen immunotherapy preparation concentration of 10,000 TU/mL; the administration protocol consists of 0.2 mL and 0.3 mL subcutaneously, 30 min apart. The patient is observed for 30 min between doses and for one hour after the second injection for possible systemic allergic reactions. This allergen-specific immunotherapy protocol is considered to achieve a maintenance dose of 0.5 mL subcutaneously within one day. The Rush method is applied according to the manufacturer’s protocol. Subsequent doses of ASIT are administered at intervals of 4–6 weeks. The schedule of sample collection for laboratory analysis and questionnaire completion is presented in Figure 1.

### 2.5. Statistical Analysis

Statistical analyses were performed using IBM SPSS Statistics for Windows (version 29.0.2.0; SPSS Inc., Chicago, IL, USA). As the differences in RQLQ scores did not significantly deviate from normality (Shapiro–Wilk *p* > 0.05), pre- and post-treatment differences were assessed using a paired *t*-test (except for eye symptoms, where the non-parametric Wilcoxon signed-rank test was used). The data for sIgE against HDM components met the normality criterion; however, given the small study sample, we used non-parametric criteria for comparison and compared medians. Correlations (Spearman’s rho) were used to examine relationships between two quantitative variables (vitamin D, IgE concentration, or BMI). Results were considered statistically significant when *p* < 0.05 for all analyses.

## 3. Results

### 3.1. Subjects’ Demographic Data and Vitamin D and IgE Levels

Seven participants, of whom five were diagnosed with allergic rhinitis and two with allergic rhinitis and allergic asthma, were included in this pilot study. Six participants were men, and one was a woman. All the participants were sensitized to house dust mite allergens and did not have sensitization to other allergens. Their ages ranged from 24 to 42 years (mean: 32.42) (see Table 1). A trend was observed in which lower concentrations of vitamin D before ASIT treatment were associated with higher IgE concentrations (Kendall’s tau b = −0.619, *p* = 0.051). No associations between BMI and vitamin D or IgE concentrations in serum were identified.

### 3.2. Dynamics of Allergen-Specific IgE Levels During Allergen-Specific Immunotherapy

**Figure 2:**
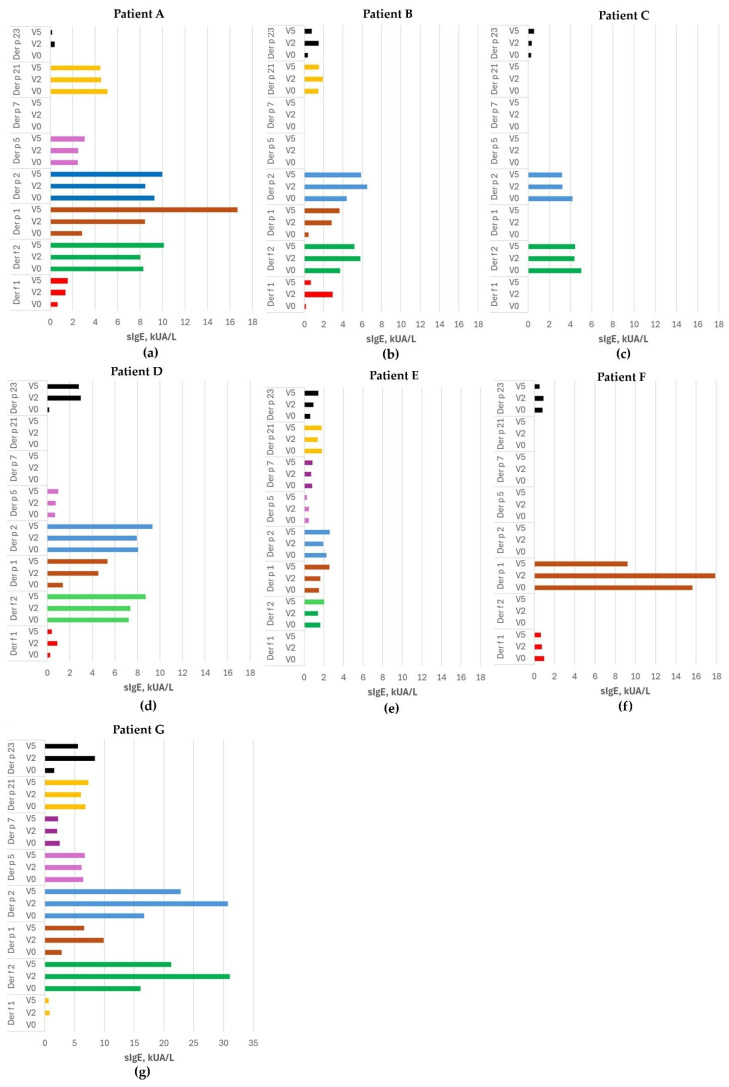
The dynamics of sIgE against HDM allergen components during ASIT treatment. The sensitization profiles for each subject are shown in (**a**–**g**). Abbreviations: sIgE—specific immunoglobulin E.

The results of sIgE dynamics against *D. pteronyssinus* and *D. farinae* allergens for each patient (A–G) are presented in Figure 2. Participants were sensitized from three to eight allergen components of HDM, of which the most prevalent were Der f 2 and Der p 1, 2, and 23. No consistent decrease or increase in sIgE concentration was observed. The sIgE profile varied individually; most often, after the introduction of ASIT (V2), sIgE increased and then decreased after the third maintenance dose (V5). However, a statistically significant increase in median sIgE concentration after the introduction of ASIT was observed for the Der p 1 and Der p 23 components (*p* = 0.028 and 0.018, respectively). Interestingly, patients who were not sensitized to the Der p 23 component before treatment developed de novo sensitization to the Der p 23 component after the introduction of ASIT (patients A and D). Furthermore, the median sIgE level for the Der p 23 component increased significantly between visits V0 and V5 (see Table 2). No significant dynamics in sIgE concentrations against other HDM allergen components were observed between visits V0 and V5 (*p* > 0.05). Additionally, no significant changes in total IgE were observed between visits V0 and V2, V2 and V5, or V0 and V5 (*p* > 0.05).

### 3.3. Life Quality Changes During Treatment

**Figure 3:**
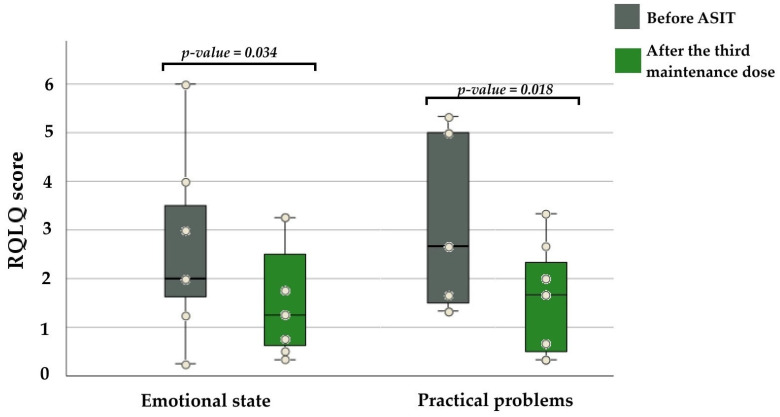
Improved quality of life indicators after the third maintenance dose of ASIT treatment. Abbreviations: RQLQ—Rhinoconjunctivitis Quality of Life Questionnaire.

To assess the role of ASIT in improving patients’ quality of life, the Rhinoconjunctivitis Quality of Life Questionnaire (RQLQ) and total nasal symptom score (TNSS) were calculated, providing an objective assessment of subjective symptom changes (see Table 3). Quality-of-life indicators did not change significantly during the ASIT induction period (V0 to V2). After the third maintenance dose, patients reported improvement in their emotional state and encountered fewer practical problems in daily activities. RQLQ scores for these two aspects decreased significantly (*p* = 0.034; 0.018), indicating a positive effect of ASIT on subjective well-being (see Figure 3). No significant changes were observed in the remaining RQLQ domains. TNSS scores did not differ significantly during the reporting period (*p* = 0.588). The median TNSS score at V0 was 5 (min 2–max 8), and at V5 it was 4 (min 3–max 7).

## 4. Discussion

This pilot study aimed to demonstrate the dynamics of sIgE concentrations against house dust mite allergen components during allergen-specific immunotherapy and to assess changes in patients’ total nasal score and quality of life. We were the first to show that, after initiating ASIT treatment, there is a significant increase in sIgE against the Der p 23 component. Furthermore, changes in patients’ quality of life occur no earlier than after the third maintenance dose of ASIT. Additionally, a tendency was observed whereby lower vitamin D concentrations before ASIT treatment were associated with higher total IgE concentrations. However, no associations between BMI and vitamin D or total IgE concentrations in serum were found.

Researchers are seeking biomarkers to personalize ASIT treatment or assess its effectiveness. Alterations in sIgE depend on the influence of regulatory T lymphocytes via the induction of IgG4 [29,30]. As a result, IgE-mediated allergic reactions are inhibited by the competitive binding of IgG4 to the allergen. Due to the long-term effects of immunotherapy, the ratio of sIgE and sIgE/sIgG4 decreases, while the amount of sIgG4 increases [20,31]. However, the temporal dynamics of sIgE levels remain incompletely elucidated. If these immunologic changes were objectively defined, ASIT efficacy could be assessed using biologically grounded measures rather than relying solely on patient-reported outcomes such as quality-of-life questionnaires and TNSS scores, as is currently the case. According to the results of our pilot study, in most cases, after the introduction of ASIT, sIgE initially increased, and then decreased after the third maintenance dose. The observed decrease in sIgE suggests a shift in the immune response from a dominant type 2 to a more regulatory profile, as described in previous ASIT studies [32,33,34]. A statistically significant increase in median sIgE concentration after the introduction of ASIT was observed against the Der p 1 and Der p 23 components. Furthermore, the concentration of sIgE against the Der p 23 component increased in all patients: those who were sensitized to this component before treatment, as well as patients who were not sensitized to the Der p 23 component before therapy and developed de novo sensitization after treatment initiation. From a mechanistic perspective, no new sensitizations should occur during ASIT treatment; however, clinical data are inconclusive. It is important to understand that the “development of new sensitizations” may depend not only on therapy but also on how intensively sensitization was studied, which methods were used, which allergens were monitored, and the duration of the monitoring period. In addition, ASIT may cause temporary sensitization to the therapeutic preparation itself (i.e., the extract with which the patient is treated) [35]. This may not represent a new sensitization to another allergen, but rather the dynamics of the immune response to the treatment antigen. Nevertheless, data show that after 3 years of ASIT treatment against pollen or house dust mites, 20% of patients developed de novo sensitization [36]. Our findings regarding increasing Der p 23 levels during ASIT treatment are essential in light of current studies examining the clinical relevance of Der p 23. In mouse experimental models, recombinant Der p 23 (rDer p 23) elicits strong type-2–biased immune responses and induces marked allergic airway inflammation in vivo, including eosinophilic and neutrophilic infiltration. Moreover, serum IgE reactivity to rDer p 23 was approximately two-thirds higher in *D. pteronyssinus*-sensitized asthmatic individuals, and both basophil activation and skin prick responses were significantly elevated compared with controls. These findings support the strong pro-allergenic potential of Der p 23 and underline its importance as a clinically relevant HDM component [37].

Currently, the RQLQ and TNSS questionnaires, which are partly subjective assessments, are used as the main indicators for evaluating the effectiveness of ASIT. Our results showed that a reduction in emotional and practical problems was significantly noticeable as early as the third maintenance dose. However, the TNSS score did not decrease significantly. The discrepancy between these questionnaires indicates that subjective improvements in quality of life do not always directly correspond to objective symptomatic changes. This highlights another problematic aspect of ASIT treatment—the placebo effect. Improvement in RQLQ scores may reflect both true subjective symptom relief and a psychological mediated placebo effect; that is, patients may feel better due to increased confidence in the treatment or perceived attention to their condition. By contrast, the TNSS questionnaire is more symptomatic and physiologically oriented (e.g., nasal congestion, sneezing, rhinorrhea) and is less susceptible to placebo effects; therefore, its stability indicates that objective inflammatory activity has persisted. Even if the placebo effect contributed in part, such changes still have practical importance for patients. The placebo effect in ASIT treatment was highlighted by the EAACI working group, which noted that the placebo effect in ASIT trials may be particularly strong for subjective indicators such as RQLQ, where patient expectations, investigator–patient interactions, and treatment context play an important role [38]. Nevertheless, the effectiveness of ASIT is not in doubt [39,40]. Evidence suggests that one year of subcutaneous ASIT significantly improves quality of life in patients with AR [41]. However, results regarding TNSS score improvement during ASIT treatment are controversial. Some studies report that TNSS score improves greatly after one year of allergen immunotherapy [42,43,44], whereas others found that the TNSS score decreases after three months of ASIT treatment, with no further changes observed between months 6 and 12 of treatment [45]. Data analysis suggests that variations in study design and monitoring frequency may lead to different conclusions regarding changes in TNSS scores during ASIT.

Immune biomarkers such as sIgE provide insight into sensitization profiles And may serve as valuable biomarkers for evaluating the effectiveness of ASIT. Still, other individual factors may also influence immune response modulation and treatment outcomes, such as vitamin D status and BMI. For example, studies in large populations have shown that vitamin D levels are inversely correlated with IgE levels [46]. In addition, obesity has been shown to increase in parallel with allergic diseases, and vitamin D deficiency is associated with higher BMI [46,47]. It has been hypothesized that vitamin D may improve glucose metabolism [48,49]. Despite the small sample size of our study, we also observed a tendency for lower vitamin D concentrations to be associated with higher IgE concentrations. However, we were unable to establish a link between vitamin D and BMI. Data indicate that vitamin D levels differ by gender [50,51]. Scientists studying vitamin D levels and BMI suggest that increasing adiposity leads to resistance to vitamin D, such that vitamin D concentrations in individuals with higher BMI do not increase as much as in those with lower BMI [52,53]. Women generally have higher fat mass, which may explain the observed lower vitamin D levels and the stronger relationship between lower vitamin D and higher BMI [51]. Our study group consisted mainly of men, this may explain why we did not find a significant correlation between vitamin D and BMI.

In this context, our findings are consistent with those of other researchers and strengthen the hypothesis that ASIT studies should combine both subjective and objective assessment methods to distinguish specific immunotherapy effects from non-specific placebo and contextual factors. Therefore, identifying biomarkers whose dynamics during treatment can help assess the objective effectiveness of ASIT treatment is crucial.

This study has certain limitations. The observation period was limited to 6 months, and the sample size was relatively small. However, our primary objective was to investigate changes in allergen sensitization profile during the initial phase of treatment, since data on this early period are scarce. Thus, we believe that our pilot study offers novel insights into the early dynamics of allergen-specific immunotherapy.

## 5. Conclusions

After six months of ASIT treatment against house dust mite allergens, a statistically significant increase in sIgE against the Der p 23 component was observed. Therefore, continuous monitoring of this component throughout the remaining stages of ASIT is essential to determine whether these changes reflect de novo sensitization or an immunological response to therapy. Quality-of-life evaluation revealed that after six months of ASIT treatment, patients experienced a significant reduction in practical problems and an improvement in emotional well-being, while nasal symptom scores remained unchanged.

## Figures and Tables

**Table 1:** Description of the study group, including laboratory test results. Abbreviations: AR—allergic rhinitis, AA—allergic asthma, BMI—body mass index.

Patient	Gender	Age	Diagnosis	Concentration of Vit. D, nmol/L	Concentration of Total IgE, kU/L *	BMI
A	Male	24	AR + AA	47.00	23.50	24.20
B	Male	42	AR	73.20	159.20	28.80
C	Male	26	AR	95.20	135.40	32.10
D	Female	27	AR	102.70	10.70	23.00
E	Male	32	AR + AA	28.50	192.50	40.40
F	Male	35	AR	107.70	99.00	26.00
G	Male	41	AR	42.10	179.60	15.70

* The concentration of total IgE before ASIT treatment.

**Table 2:** Comparison of median sIgE levels (kUA/L) at baseline (V0) and during the treatment (V5) of ASIT against house dust mite allergen components. Abbreviations: V—visit number.

HDM Allergen Component	Visit No.	Median [Min–Max]	*p*-Value		
			V0–V2	V2–V5	V0–V5
Der p 1	V0	2.16 [0.43–15.64]	0.028 *		-
	V2	6.48 [0.43–15.64]		0.917	-
	V5	5.96 [2.58–16.68]	-		0.249
Der p 2	V0	6.23 [2.28–16.70]	0.917		
	V2	7.24 [1.93–30.74]		0.753	-
	V5	7.6 [2.61–22.79]	-		0.116
Der p 5	V0	1.57 [0.47–6.45]	0.655		-
	V2	1.59 [0.47–6.15]		0.144	-
	V5	2.00 [0.29–6.73]	-		0.144
Der p 7	V0	1.67 [0.82–2.51]	0.18		-
	V2	1.67 [ 0.70–2.09]		0.180	-
	V5	1.52 [0.83–2.21]	-		0.655
Der p 21	V0	3.45 [1.46–6.81]	0.273	-	-
	V2	3.21 [1.37–6.04]		0.465	-
	V5	3.12 [1.52–7.33]	-		1
Der p 23	V0	0.37 [0.00–1.56]	0.018 *	-	-
	V2	0.95 [0.34–8.39]		0.310	-
	V5	0.77 [0.15–5.56]	-		0.046 *
Der f 1	V0	0.25 [0.11–0.98]	0.08	-	-
	V2	0.88 [ 0.74–2.92]		0.225	-
	V5	0.65 [0.40–1.51]	-		0.138
Der f 2	V0	6.13 [1.63–16.06]	0.753	-	-
	V2	6.59 [1.42–31.07]		0.753	-
	V5	6.96 [2.03–21.22]	-		0.075

* Statistically significant.

**Table 3:** Subjective assessment of quality of life of study participants during ASIT treatment. RQLQ scores are indicated in the table.

Domain	Visit No.	Median	MIN–MAX	*p*-Value
Daily activities	V0	3	2–5.33	0.09
	V5	2	0.67–5.00	
Sleeping state	V0	1.67	0.00–4.67	0.528
	V5	1.67	0.67–2.33	
Eye symptoms	V0	3.5	0.00–4.25	0.068
	V5	1.5	0.00–4.00	
Non-nasal/eye symptoms	V0	2.23	0.29–3.00	0.753
	V5	1.43	0.67–3.29	
Practical problems	V0	2.67	1.33–5.33	0.018
	V5	1.67	0.33–3.33	
Emotional state	V0	2	0.25–6.00	0.034
	V5	1.25	0.33–3.25	
Nasal symptoms	V0	3	1.00–5.00	0.203
	V5	2	0.33–3.25	

## Data Availability

The original contributions presented in this study are included in the article. Further inquiries can be directed to the corresponding author.

## Abbreviations

The following abbreviations are used in this manuscript:

ASIT	Allergen-specific immunotherapy
BMI	Body Mass Index
sIgE	Specific immunoglobulin E
RQLQ	Rhinoconjunctivitis Quality of Life Questionnaire
SCIT	Subcutaneous Immunotherapy
TNSS	Total Nasal Symptom Score
